# This is not the end: A long‐awaited update of the definition of pain

**DOI:** 10.1002/pne2.12058

**Published:** 2021-08-05

**Authors:** Randi Dovland Andersen

One of the first things I learned when becoming a researcher was the importance of defining the terms I used. Different disciplines attribute different meanings to identical terms. The first example that comes to mind is qualitative data. A qualitative researcher will understand it to mean unstructured data collected through methods such as observations or interviews,^1^ while a quantitative researcher may use the term to refer to categorical data.^2^ If we understand the terms we use differently, communication will be difficult. Definitions are important because they provide a common language and enable a common or shared understanding of a phenomenon.

In June 2020, the International Association of Pain (IASP) presented a revision to their well‐known 1979 definition of pain. The revised definition describes pain as “*an unpleasant sensory and emotional experience associated with, or resembling that associated with, actual or potential tissue damage*”.^3^ A 14‐member multi‐national Task Force, including several members from the pediatric pain research community, led by Dr. Srinivasa N. Raja from the Johns Hopkins University School of Medicine had developed the revision. The Task Force described the revision process and presented the new definition and its accompanying notes in an article in PAIN.^4^ There is also an infographic or graphical abstract available.

In this special section, we have invited researchers and individuals with lived experience to share their thoughts on the revised definition. It was important for us to provide a platform to continue the ongoing scientific discussion concerning the definition of pain, which led to its revision. The revised definition may have addressed some of the controversies raised over the past decades; see, for example, Ref. 5, 6, 7, 8 while others may remain.

Dr. Bonnie Stevens, widely recognized for her work on pain in neonates, was one of the pediatric pain researchers on the IASP Task Force. In her commentary, she outlines the implications of the revised definition on our understanding of pain in children, in particular those who are nonverbal.^9^ One of the major criticisms toward the previous definition was its emphasis on the individuals’ ability to describe their pain. Dr. Stevens finds the revised definition with its accompanying notes more beneficial to assessment of pain in children because it acknowledges other indicators of pain besides self‐report.

For decades, Dr. Ken Craig has contributed to the debate concerning the definition of pain. Together with Kanwaljeet (Sunny) Anand, he early addressed its exclusion of nonverbal populations,^7^ which led to a change in the accompanying notes. Later, his work has emphasized social and cognitive aspects of the experience of pain,^10^, ^11^, ^12^ including how these aspects were missing from the definition.^8^ In their review, Ken Craig and Nicole MacKenzie review the evidence for social and cognitive factors in the experience of pain and with this lens explore the revised definition and its accompanying notes. They argue that the new definition continue to support a narrow biomedical model, despite advances in interdisciplinary care, patient engagement, and the effectiveness and use of psychological interventions to manage pain.^13^

Finally, how we define pain is not only important to clinicians and researchers. Individuals living with pain and their family members are influenced by how their providers understand, evaluate, manage, and communicate about pain. In the commentary by Isabel Jordan, Rachel Martens, and Katie Birnie, they reflect upon how personal experience has helped shape the pediatric pain field, their own experiences, and the value of the revised definition from a patient/parent perspective. They find the revised definition more supportive of patient experiences, but emphasize that a better definition is not enough. It is merely a starting point. They issue a call for action to change how children with pain and their families are met and their pain is managed.^14^

Although we have a new, in some ways improved definition of pain, it is not perfect, and the scientific debate concerning how we understand and define pain will continue. As a community, we also need to respond to the call for action from those with lived experiences. As the only journal dedicated to pain in children and adolescents, Paediatric and Neonatal Pain encourages further submissions on these important subjects.
